# 1-[(4,5-Dimethyl­cyclo­hexa-1,4-dien-1-yl)sulfon­yl]-4-methyl­benzene

**DOI:** 10.1107/S1600536808021260

**Published:** 2008-08-06

**Authors:** Ryan H. Gray, Frank R. Fronczek, M. Graça H. Vicente

**Affiliations:** a Department of Chemistry, Louisiana State University, Baton Rouge, LA 70803-1804, USA

## Abstract

In the title mol­ecule, C_15_H_18_O_2_S, the dimethyl­cyclo­hexa­diene unit is slightly non-planar, having a folded conformation with the two double-bond planes forming a dihedral angle of 3.9 (6)°. Methyl groups of the dimethyl­cyclo­hexa­diene ring tilt away from each other, forming inter­nal C—C—C(Me) angles approximately 11° greater than the exterior angles.

## Related literature

For related literature, see: Filatov *et al.* (2007 ▶); Glidewell *et al.* (2001 ▶); Loudet & Burgess (2007 ▶); Ogura *et al.* (2001 ▶); Tanui *et al.* (2008 ▶); Ongayi (2005 ▶); Pomarico (2009 ▶).
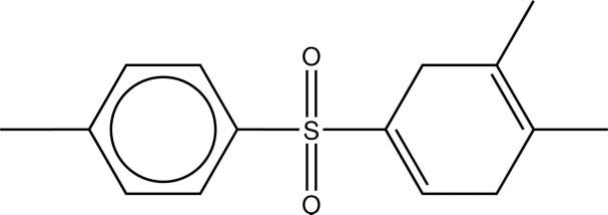


## Experimental

### 

#### Crystal data

C_15_H_18_O_2_S
*M* *_r_* = 262.35
Monoclinic, 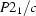

*a* = 14.1607 (10) Å
*b* = 7.5766 (5) Å
*c* = 12.6923 (10) Å
β = 101.658 (5)°
*V* = 1333.66 (17) Å^3^
*Z* = 4
Cu *K*α radiation
μ = 2.08 mm^−1^
*T* = 90 K
0.37 × 0.29 × 0.21 mm

#### Data collection

Bruker Kappa APEXII CCD area-detector diffractometer
Absorption correction: multi-scan (*SADABS*; Sheldrick, 2008 ▶) *T*_min_ = 0.513, *T*_max_ = 0.669
8185 measured reflections
2414 independent reflections
2388 reflections with *I* > 2σ(*I*)
*R*_int_ = 0.017

#### Refinement

*R*[*F* ^2^ > 2σ(*F* ^2^)] = 0.031
*wR*(*F* ^2^) = 0.079
*S* = 1.02
2414 reflections
167 parameters
H-atom parameters constrained
Δρ_max_ = 0.39 e Å^−3^
Δρ_min_ = −0.35 e Å^−3^

### 

Data collection: *APEX2* (Bruker, 2006 ▶); cell refinement: *APEX2*; data reduction: *APEX2*; program(s) used to solve structure: *SHELXS97* (Sheldrick, 2008 ▶); program(s) used to refine structure: *SHELXL97* (Sheldrick, 2008 ▶); molecular graphics: *ORTEP-3 for Windows* (Farrugia, 1997 ▶); software used to prepare material for publication: *SHELXTL* (Sheldrick, 2008 ▶).

## Supplementary Material

Crystal structure: contains datablocks global, I. DOI: 10.1107/S1600536808021260/fl2208sup1.cif

Structure factors: contains datablocks I. DOI: 10.1107/S1600536808021260/fl2208Isup2.hkl

Additional supplementary materials:  crystallographic information; 3D view; checkCIF report

## Figures and Tables

**Fig. 1.**
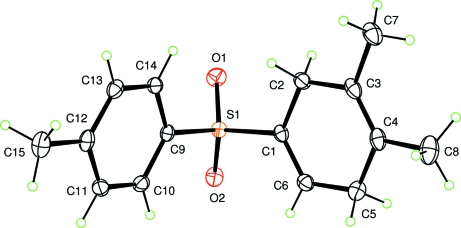
Ellipsoids at the 50% level, with H atoms having arbitrary radius.

**Fig. 2.**
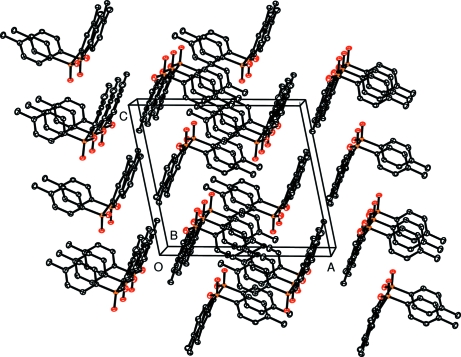
The packing viewed approximately down the symmetry direction. H atoms are not shown.

## supplementary crystallographic information

### Comment

The title compound, synonym 3,4-dimethyl-tosyl-cyclohexadiene, (I) is an important intermediate in the synthesis of 3,4-disubstituted pyrroles and isoindoles (Filatov *et al.*, 2007). In particular, the 4,7-dihydroisoindole obtained in one step from (I) has been used in the total syntheses of tetrabenzoporphyrins (TBP) and tetrabenzocorroles (TBC) for application in cancer therapy by photodynamic therapy (PDT), and in the syntheses of BODIPY-type molecules for cancer imaging and diagnosis. On account of their strong absorptions and fluorescence emissions in the near IR region of the spectrum and their high chemical stability, these compounds have shown promise for the above biomedical applications (Ongayi, 2005; Loudet & Burgess, 2007). Compound (I) was prepared by a Diels-Alder cycloaddition reaction of 2,3-dimethylbutadiene with tosyl-acetylene at 60–70 °C.

Compound (I) contains a sulfonyl center which offsets both the tolyl and the dimethylcyclohexadienyl groups from linearity, with C1—S1—C9 angle 102.96 (6)°. Similarly, a 104.20 (5)° C—S—N angle is found in a related tosyl-pyrrole (Tanui *et al.*, 2008).

The cyclohexadiene ring in (I) is nearly planar, exhibiting a slight fold along the C2···C5 line, which joins the two C—C==C—C planes. Those planes form a dihedral angle of 3.9 (6)°. The cyclohexadiene ring forms a dihedral angle of 85.70 (3)° with the phenyl ring plane. The two methyl groups on adjacent C atoms of the cyclohexadiene ring are bent away from each other, causing the interior C—C—C(Me) angles to be approximately 11° greater than exterior angles. The interior angles are 124.36 (15)° at C3 and 123.76 (15)° at C4, while the exterior angles are 112.90 (14)° at C3 and 113.97 (14)°. The methyl groups also twist out of plane, forming a C7—C3—C4—C8 torsion angle of 2.1 (2)°. These methyl groups have an intramolecular H···H contact 2.08 Å (based on H positions determined with HFIX 137), about 0.3 Å less than their van der Waals radii sum.

The structure of a related tosylate compounds have been reported, *i.e.* 3,4-dimethylbenzenesulfonyl chloride (Glidewell *et al.*, 2001) and 3,4,4-trimethyldiphenyl sulfone (Ogura *et al.*, 2001). The first, QIBREY, differs in 2 ways: a 3,4-dimethylphenyl rather than a 3,4-dimethylcyclohexadiene and a *p*-Cl phenyl rather than tolyl on the SO_2_ center. QIBREY has very similar cell dimensions (in its *P*2_1_/*n* setting) to (I), and also similar packing, but the two structures are not isomorphous.

### Experimental

The synthesis of the compound was adapted from Pomarico (2009): To a 50 ml round bottom flask, tosyl-acetylene (MW 180, 1.3305 g, 7.39 mmol) and 832 υl of 2,3-dimethylbutadiene (MW 82.15, d 0.726 g/ml) were dissolved in 25 ml of anhydrous toluene. N_2_ was introduced for an inert atmosphere. Reaction tube was stirred at 60–70° C for 72 h. Residue was purified by a silica gel column and eluted with hexane-ethyl acetate (4:1). (I) was obtained in the first band. The desired fractions were covered with Parafilm and punctured to allow solvent evaporation. After 72 h, the most concentrated fraction(*s*) was driven to super-saturation and formed needle crystals (compound I) in solution. The other fractions yielded a yellow-white solid powder (compound I, impure). Yield: 71% (1.3864 g, 5.27 mmol). Spectroscopic analysis, ^1^H NMR (400 MHz, CDCl_3_, 299 K): 7.78 (2*H*, d, CH), 7.37 (2*H*, d, CH), 6.93 (1*H*, s, CH), 2.80 (2*H*, s, CH_2_), 2.65 (2*H*, d, CH_2_), 2.39 (3*H*, s, CH_3_), 1.57 (6*H*, s, CH_3_). MS (EI) m/*z*: 263.1098 (*M*^+^).

### Refinement

H atoms on C were placed in idealized positions with C—H distances 0.95 - 0.99 Å and thereafter treated as riding. *U*_iso_ for H was assigned as 1.2 times *U*_eq_ of the attached atoms (1.5 for methyl).

### Crystal data

**Table:**

C_15_H_18_O_2_S	*F*_000_ = 560
*M**_r_* = 262.35	*D*_x_ = 1.307 Mg m^−^^3^
Monoclinic, *P*2_1_/*c*	Cu *K*α radiation λ = 1.54178 Å
Hall symbol: -P 2ybc	Cell parameters from 6824 reflections
*a* = 14.1607 (10) Å	θ = 3.1–69.6º
*b* = 7.5766 (5) Å	µ = 2.08 mm^−^^1^
*c* = 12.6923 (10) Å	*T* = 90 K
β = 101.658 (5)º	Needle fragment, colourless
*V* = 1333.66 (17) Å^3^	0.37 × 0.29 × 0.21 mm
*Z* = 4	

### Data collection

**Table:**

Bruker Kappa APEXII CCD area-detector diffractometer	2414 independent reflections
Radiation source: fine-focus sealed tube	2388 reflections with *I* > 2σ(*I*)
Monochromator: graphite	*R*_int_ = 0.017
*T* = 90 K	θ_max_ = 70.0º
φ and ω scans	θ_min_ = 6.3º
Absorption correction: multi-scan(SADABS; Sheldrick, 2008)	*h* = −16→17
*T*_min_ = 0.513, *T*_max_ = 0.669	*k* = −8→8
8185 measured reflections	*l* = −14→15

### Refinement

**Table:**

Refinement on *F*^2^	Hydrogen site location: inferred from neighbouring sites
Least-squares matrix: full	H-atom parameters constrained
*R*[*F*^2^ > 2σ(*F*^2^)] = 0.031	*w* = 1/[σ^2^(*F*_o_^2^) + (0.0349*P*)^2^ + 1.1601*P*] where *P* = (*F*_o_^2^ + 2*F*_c_^2^)/3
*wR*(*F*^2^) = 0.079	(Δ/σ)_max_ = 0.001
*S* = 1.02	Δρ_max_ = 0.39 e Å^−^^3^
2414 reflections	Δρ_min_ = −0.35 e Å^−^^3^
167 parameters	Extinction correction: SHELXL97 (Sheldrick, 2008), Fc^*^=kFc[1+0.001xFc^2^λ^3^/sin(2θ)]^-1/4^
Primary atom site location: structure-invariant direct methods	Extinction coefficient: 0.0014 (2)
Secondary atom site location: difference Fourier map	

### Special details

**Table:**

Geometry. All e.s.d.'s (except the e.s.d. in the dihedral angle between two l.s. planes) are estimated using the full covariance matrix. The cell e.s.d.'s are taken into account individually in the estimation of e.s.d.'s in distances, angles and torsion angles; correlations between e.s.d.'s in cell parameters are only used when they are defined by crystal symmetry. An approximate (isotropic) treatment of cell e.s.d.'s is used for estimating e.s.d.'s involving l.s. planes.
Refinement. Refinement of *F*^2^ against ALL reflections. The weighted *R*-factor *wR* and goodness of fit *S* are based on *F*^2^, conventional *R*-factors *R* are based on *F*, with *F* set to zero for negative *F*^2^. The threshold expression of *F*^2^ > σ(*F*^2^) is used only for calculating *R*-factors(gt) *etc*. and is not relevant to the choice of reflections for refinement. *R*-factors based on *F*^2^ are statistically about twice as large as those based on *F*, and *R*- factors based on ALL data will be even larger.

### Fractional atomic coordinates and isotropic or equivalent isotropic displacement parameters (Å^2^)

**Table:**

	*x*	*y*	*z*	*U*_iso_*/*U*_eq_	
S1	0.72081 (2)	0.65111 (5)	0.77242 (3)	0.01466 (12)	
O1	0.77069 (8)	0.81816 (14)	0.78620 (9)	0.0217 (3)	
O2	0.69509 (8)	0.57537 (14)	0.66619 (8)	0.0203 (2)	
C1	0.78977 (10)	0.4965 (2)	0.85979 (11)	0.0150 (3)	
C2	0.84519 (10)	0.5644 (2)	0.96471 (12)	0.0181 (3)	
H2A	0.8013	0.6342	1.0001	0.022*	
H2B	0.8964	0.6447	0.9507	0.022*	
C3	0.89062 (10)	0.4216 (2)	1.04018 (12)	0.0192 (3)	
C4	0.88460 (10)	0.2504 (2)	1.01358 (12)	0.0199 (3)	
C5	0.83462 (11)	0.1878 (2)	0.90446 (12)	0.0213 (3)	
H5A	0.8827	0.1294	0.8695	0.026*	
H5B	0.7863	0.0978	0.9137	0.026*	
C6	0.78555 (10)	0.3272 (2)	0.83147 (12)	0.0172 (3)	
H6	0.7501	0.2946	0.7625	0.021*	
C7	0.93992 (11)	0.4914 (2)	1.14845 (12)	0.0258 (4)	
H7A	0.8916	0.5402	1.1857	0.039*	
H7B	0.9856	0.5841	1.1388	0.039*	
H7C	0.9746	0.3951	1.1913	0.039*	
C8	0.92390 (13)	0.1033 (3)	1.09036 (14)	0.0301 (4)	
H8A	0.9855	0.1401	1.1352	0.045*	
H8B	0.9339	−0.0023	1.0494	0.045*	
H8C	0.8779	0.0768	1.1364	0.045*	
C9	0.61462 (10)	0.67078 (18)	0.82478 (11)	0.0138 (3)	
C10	0.53295 (10)	0.57652 (19)	0.77775 (12)	0.0171 (3)	
H10	0.5326	0.5072	0.7153	0.021*	
C11	0.45159 (10)	0.58476 (19)	0.82322 (12)	0.0185 (3)	
H11	0.3950	0.5229	0.7903	0.022*	
C12	0.45178 (11)	0.6823 (2)	0.91624 (12)	0.0178 (3)	
C13	0.53499 (11)	0.7747 (2)	0.96241 (11)	0.0177 (3)	
H13	0.5361	0.8410	1.0262	0.021*	
C14	0.61613 (10)	0.77178 (19)	0.91700 (11)	0.0157 (3)	
H14	0.6719	0.8375	0.9482	0.019*	
C15	0.36432 (12)	0.6882 (2)	0.96662 (13)	0.0248 (4)	
H15A	0.3324	0.8029	0.9519	0.037*	
H15B	0.3841	0.6716	1.0445	0.037*	
H15C	0.3196	0.5940	0.9363	0.037*	

### Atomic displacement parameters (Å^2^)

**Table:**

	*U*^11^	*U*^22^	*U*^33^	*U*^12^	*U*^13^	*U*^23^
S1	0.01540 (19)	0.0156 (2)	0.01337 (19)	0.00057 (13)	0.00388 (13)	0.00124 (13)
O1	0.0225 (6)	0.0181 (5)	0.0260 (6)	−0.0034 (4)	0.0087 (4)	0.0027 (4)
O2	0.0232 (5)	0.0250 (6)	0.0128 (5)	0.0045 (4)	0.0039 (4)	0.0005 (4)
C1	0.0121 (6)	0.0188 (7)	0.0144 (7)	0.0010 (6)	0.0034 (5)	0.0008 (6)
C2	0.0149 (7)	0.0216 (8)	0.0172 (7)	0.0000 (6)	0.0018 (6)	−0.0032 (6)
C3	0.0123 (7)	0.0312 (9)	0.0144 (7)	0.0017 (6)	0.0037 (5)	−0.0014 (6)
C4	0.0153 (7)	0.0277 (9)	0.0175 (7)	0.0048 (6)	0.0055 (6)	0.0029 (6)
C5	0.0217 (8)	0.0199 (8)	0.0226 (8)	0.0034 (6)	0.0050 (6)	0.0005 (6)
C6	0.0152 (7)	0.0207 (8)	0.0155 (7)	−0.0004 (6)	0.0030 (6)	−0.0018 (6)
C7	0.0205 (8)	0.0399 (10)	0.0161 (7)	0.0047 (7)	0.0013 (6)	−0.0043 (7)
C8	0.0313 (9)	0.0366 (10)	0.0236 (8)	0.0125 (8)	0.0086 (7)	0.0093 (7)
C9	0.0147 (7)	0.0127 (7)	0.0141 (7)	0.0020 (5)	0.0033 (5)	0.0029 (5)
C10	0.0196 (7)	0.0139 (7)	0.0168 (7)	0.0017 (6)	0.0015 (6)	−0.0002 (6)
C11	0.0156 (7)	0.0143 (7)	0.0244 (8)	−0.0011 (5)	0.0012 (6)	0.0026 (6)
C12	0.0189 (7)	0.0151 (7)	0.0199 (7)	0.0051 (6)	0.0055 (6)	0.0084 (6)
C13	0.0219 (7)	0.0169 (7)	0.0141 (7)	0.0050 (6)	0.0031 (6)	0.0013 (6)
C14	0.0166 (7)	0.0140 (7)	0.0154 (7)	0.0014 (5)	0.0002 (5)	0.0007 (6)
C15	0.0224 (8)	0.0252 (8)	0.0292 (9)	0.0051 (6)	0.0110 (7)	0.0089 (7)

### Geometric parameters (Å, °)

**Table:**

S1—O1	1.4427 (11)	C7—H7C	0.9800
S1—O2	1.4427 (11)	C8—H8A	0.9800
S1—C1	1.7656 (14)	C8—H8B	0.9800
S1—C9	1.7687 (14)	C8—H8C	0.9800
C1—C6	1.331 (2)	C9—C10	1.387 (2)
C1—C2	1.4939 (19)	C9—C14	1.395 (2)
C2—C3	1.501 (2)	C10—C11	1.390 (2)
C2—H2A	0.9900	C10—H10	0.9500
C2—H2B	0.9900	C11—C12	1.392 (2)
C3—C4	1.339 (2)	C11—H11	0.9500
C3—C7	1.506 (2)	C12—C13	1.394 (2)
C4—C5	1.499 (2)	C12—C15	1.505 (2)
C4—C8	1.511 (2)	C13—C14	1.386 (2)
C5—C6	1.482 (2)	C13—H13	0.9500
C5—H5A	0.9900	C14—H14	0.9500
C5—H5B	0.9900	C15—H15A	0.9800
C6—H6	0.9500	C15—H15B	0.9800
C7—H7A	0.9800	C15—H15C	0.9800
C7—H7B	0.9800		
			
O1—S1—O2	119.15 (7)	C3—C7—H7C	109.5
O1—S1—C1	108.10 (7)	H7A—C7—H7C	109.5
O2—S1—C1	109.04 (7)	H7B—C7—H7C	109.5
O1—S1—C9	108.25 (7)	C4—C8—H8A	109.5
O2—S1—C9	108.14 (7)	C4—C8—H8B	109.5
C1—S1—C9	102.96 (6)	H8A—C8—H8B	109.5
C6—C1—C2	124.03 (13)	C4—C8—H8C	109.5
C6—C1—S1	118.76 (11)	H8A—C8—H8C	109.5
C2—C1—S1	117.11 (11)	H8B—C8—H8C	109.5
C1—C2—C3	113.62 (13)	C10—C9—C14	120.90 (13)
C1—C2—H2A	108.8	C10—C9—S1	119.54 (11)
C3—C2—H2A	108.8	C14—C9—S1	119.43 (11)
C1—C2—H2B	108.8	C9—C10—C11	119.14 (13)
C3—C2—H2B	108.8	C9—C10—H10	120.4
H2A—C2—H2B	107.7	C11—C10—H10	120.4
C4—C3—C2	122.71 (13)	C10—C11—C12	121.07 (14)
C4—C3—C7	124.36 (15)	C10—C11—H11	119.5
C2—C3—C7	112.90 (14)	C12—C11—H11	119.5
C3—C4—C5	122.24 (14)	C11—C12—C13	118.66 (14)
C3—C4—C8	123.76 (15)	C11—C12—C15	121.03 (14)
C5—C4—C8	113.97 (14)	C13—C12—C15	120.31 (14)
C6—C5—C4	115.24 (13)	C14—C13—C12	121.22 (14)
C6—C5—H5A	108.5	C14—C13—H13	119.4
C4—C5—H5A	108.5	C12—C13—H13	119.4
C6—C5—H5B	108.5	C13—C14—C9	118.98 (13)
C4—C5—H5B	108.5	C13—C14—H14	120.5
H5A—C5—H5B	107.5	C9—C14—H14	120.5
C1—C6—C5	121.90 (14)	C12—C15—H15A	109.5
C1—C6—H6	119.0	C12—C15—H15B	109.5
C5—C6—H6	119.0	H15A—C15—H15B	109.5
C3—C7—H7A	109.5	C12—C15—H15C	109.5
C3—C7—H7B	109.5	H15A—C15—H15C	109.5
H7A—C7—H7B	109.5	H15B—C15—H15C	109.5
			
O1—S1—C1—C6	−150.24 (12)	C4—C5—C6—C1	3.0 (2)
O2—S1—C1—C6	−19.33 (14)	O1—S1—C9—C10	147.20 (11)
C9—S1—C1—C6	95.35 (13)	O2—S1—C9—C10	16.82 (13)
O1—S1—C1—C2	33.22 (12)	C1—S1—C9—C10	−98.50 (12)
O2—S1—C1—C2	164.13 (10)	O1—S1—C9—C14	−36.97 (13)
C9—S1—C1—C2	−81.20 (12)	O2—S1—C9—C14	−167.34 (11)
C6—C1—C2—C3	−4.3 (2)	C1—S1—C9—C14	77.34 (12)
S1—C1—C2—C3	172.03 (10)	C14—C9—C10—C11	0.5 (2)
C1—C2—C3—C4	2.4 (2)	S1—C9—C10—C11	176.30 (11)
C1—C2—C3—C7	−175.74 (12)	C9—C10—C11—C12	−1.6 (2)
C2—C3—C4—C5	2.1 (2)	C10—C11—C12—C13	1.1 (2)
C7—C3—C4—C5	−179.99 (14)	C10—C11—C12—C15	−178.78 (13)
C2—C3—C4—C8	−175.78 (14)	C11—C12—C13—C14	0.5 (2)
C7—C3—C4—C8	2.1 (2)	C15—C12—C13—C14	−179.66 (13)
C3—C4—C5—C6	−4.9 (2)	C12—C13—C14—C9	−1.5 (2)
C8—C4—C5—C6	173.25 (13)	C10—C9—C14—C13	1.0 (2)
C2—C1—C6—C5	1.6 (2)	S1—C9—C14—C13	−174.79 (11)
S1—C1—C6—C5	−174.69 (11)		
